# Impact of Electronic Alternatives to Tobacco Cigarettes on Indoor Air Particular Matter Levels

**DOI:** 10.3390/ijerph17082947

**Published:** 2020-04-24

**Authors:** Carmela Protano, Maurizio Manigrasso, Vittoria Cammalleri, Giuseppe Biondi Zoccai, Giacomo Frati, Pasquale Avino, Matteo Vitali

**Affiliations:** 1 Department of Public Health and Infectious Diseases, Sapienza University of Rome, 00185 Rome, Italy; vittoria.cammalleri@uniroma1.it (V.C.); matteo.vitali@uniroma1.it (M.V.); 2 Department of Technological Innovations, National Institute for Insurance against Accidents at Work (INAIL), via IV Novembre 144, I-00187 Rome, Italy; m.manigrasso@inail.it; 3 Department of Medico-Surgical Sciences and Biotechnologies, Sapienza University of Rome, Corso della Repubblica 74, 04100 Latina, Italy; giuseppe.biondizoccai@uniroma1.it (G.B.Z.); giacomo.frati@uniroma1.it (G.F.); 4 Mediterranea Cardiocentro, 80122 Naples, Italy; 5 IRCCS NEUROMED, 86077 Pozzilli, Italy; 6 Department of Agricultural, Environmental and Food Sciences (DiAAA), University of Molise, via De Sanctis, I-86100 Campobasso, Italy; avino@unimol.it

**Keywords:** indoor air, particulate matter, electronic cigarettes, heat-not-burn products, IQOS^®^, GLO^®^, JUUL^®^, passive smoking

## Abstract

An aerosol study was carried out in a test room measuring particulate matter (PM) with an aerodynamic diameter smaller than 10, 4, 2.5 and 1 µm (PM_10_, PM_4_, PM_2.5_, PM_1_) before and during the use of electronic alternatives to tobacco cigarettes (EATC) IQOS^®^, GLO^®^, JUUL^®^, with different kinds of sticks/pods, as well as during the smoking of a conventional tobacco cigarette. The aerosol was mainly in the PM_1_ size range (>95%). All studied EATCs caused lower indoor PM_1_ concentrations than conventional tobacco cigarettes. Nevertheless, they determined a worsening of indoor-PM_1_ concentration that ranged from very mild for JUUL^®^—depending on the pod used—to considerably severe for IQOS^®^ and GLO^®^. Median values ranged from 11.00 (Iqos3 and Juul2) to 337.5 µg m^−3^ (Iqos4). The high variability of particle loadings was attributed both to the type of stick/pod used and to the different way of smoking of volunteers who smoked/vaped during the experiments. Moreover, during vaping IQOS^®^ and GLO^®^ indoor PM_1_ concentrations reach levels by far higher than outdoor concentrations that range from 14 to 21 µg m^−3^, especially during the exhalation of the smoke. From these results emerge an urgent need of a legislative regulation limiting the use of such devices in public places.

## 1. Introduction

In recent years, new forms of electronic alternatives to tobacco cigarettes (EATCs) were developed to provide an alternative to traditional cigarettes and, currently, many types of e-cigarettes (e-cigs) and heat-not-burn products (HnBP) are commercialized worldwide.

E-cigs are integrated electric devices constituted by an atomization chamber, a stainless steel shell, a lithium ion battery assembly, a smart chip with program controlled circuits and a removable cartridge containing a mixture of propylene glycol, and/or vegetable glycerin and flavor chemicals to generate an aerosol to be inhaled and, in some cases, nicotine. Because e-cigarettes do not burn tobacco, they do not produce tar and smoke [1]. These devices were introduced for the first time into the market in 2006 and, since then, their features changed noticeably. To date, there are four different generations of e-cigs according to their technological characteristics: from the first (the simplest type) to the fourth generation (the most complex type), characterized by the possibility of modifying the operating power [2].

EATCs include also the HnBPs, electronic devices that heat tobacco to a lower temperature (ranging generally between 250 and 350 °C) than traditional cigarettes (>800 °C). The result is the absence of combustion and, in theory, the absence of combustion-derived compounds. Like e-cigs, the final effect is the production of an aerosol (but not smoke) that the user inhales. There are several commercially available HnBPs, such as IQOS^®^ (produced by Philip Morris International) and GLO^®^ (produced by British American Tobacco), constituted by a charger, a holder and an electronically controlled resistance that heats tobacco sticks [3].

The so called “pod mods”, such as the popular device JUUL^®^, represent the newest category of EATCs. JUUL^®^ device, very similar to a computer flash drive in its design, presents small replaceable cartridges called “pod-mods” containing encapsulates nicotine, flavorings and other contents in liquid form. This liquid is heated to produce vapor by the device’s battery, which can be recharged through a USB port. This device differs from prior e-cigs in the chemical formulation of nicotine. Indeed, pod-mod devices contain nicotine salts, which produce more protonated nicotine at a lower pH than the free-base form of nicotine used in other e-cigs, which has a higher pH and activates nicotine sensory receptors. Consequently, nicotine results less irritating when inhaled, and it can be delivered at higher concentration to the user. A higher dose of nicotine may benefit adult smokers who are seeking to quit cigarettes, but may also promote nicotine dependence among non-smoking adolescents and young adults [4].

The popularity of EATCs has increased in recent years, both in smokers and in non-smokers [5,6,7,8], and their use has reached a “red alarming rate” among the younger age groups of the population worldwide [9]. For example, a recent study carried out in America on a large sample of individuals evidenced that JUUL^®^ is very popular and it is currently used by a not negligible percentage of teenagers and young adults (9.5% of participants aged 15–17 and 11% aged 18–21, respectively) [10,11,12].

Even if the association between the use of EATCs and adverse effects for human health is still under debate, there are several recent evidences of possible negative outcomes [13,14,15]. In addition to the possible adverse effects for active smokers of EATCs (the so-called vapers), experimental evidences demonstrate that the use of these devices worsen indoor air quality determining a typical passive exposure also called Environmental Electronic Vape (EEV) exposure [16,17,18]. The results of our previous researches [2,19,20,21] highlighted that EEV exposure to particulate matter (PM) always occurs when e-cigs are used in indoor settings. This finding is not negligible in terms of public health as PM, independently from its composition, is a well-known risk factor for human health. Indeed, scientific evidences highlighted a strong association between exposure to PM with an aerodynamic diameter smaller than 10 µm (PM_10_) and fractions with lower aerodynamic diameters and respiratory, cardiovascular, neurodegenerative diseases or cancers [22,23,24,25]. Objective data on PM emission during and after the use of EATCs in enclosed environments still lack, but they are necessary for an accurate risk assessment.

The present study was aimed to evaluate the levels of different size fractions of PM emitted into indoor air during the use of IQOS^®^, JUUL^®^ and GLO^®^, testing different sticks for IQOS^®^ and GLO^®^ and pods for JUUL^®^.

## 2. Materials and Methods

### 2.1. Experimental Tests

Levels of PM with an aerodynamic diameter smaller than 10, 4, 2.5 and 1 µm, respectively (PM_10_, PM_4_, PM_2.5_ and PM_1_) emissions of IQOS^®^, JUUL^®^ and GLO^®^ were assessed in real use conditions, using three volunteers (identified as smoker a, b and c) who were already smokers. Each tested device was smoked using all the different types of sticks/Heets or filled cartridges (pods) available on the Italian market for a total of 14 combinations, as reported in Table 1. In addition, the same experiment was performed also with a traditional cigarette (Marlboro^®^ gold).

The order in which the products were smoked/vaped was randomized. In particular, this was an open-label randomized study based on a 2-block set of 15 sessions each, for a total of 30 sessions. The randomization was performed for ensuring random and balanced use of 6 different flavors for IQOS^®^, 4 different flavors for GLO^®^ and 4 different flavors for JUUL^®^, plus 2 sessions using tobacco cigarettes for control purposes.

The randomization produced the following sequence: Glo2, Iqos3, Glo4, Iqos6, Iqos1, Glo3, Juul4, Juul3, Juul2, Iqos2, Iqos5, Marlboro, Juul1, Glo1, Iqos4, Iqos2, Marlboro, Iqos3, Glo1, Iqos5, Juul2, Juul4, Iqos1, Iqos4, Glo2, Glo4, Iqos6, Juul1, Juul3, Glo3.

Each smoking session consisted of three consecutive experiments performed with the same combination; each volunteer took part in all the sessions, smoking one of the three consecutive tests. Thus, in total, six tests were carried out for each combination (three tests for each session of the 2-block set). The data used for subsequent processing are the average of the six measurements.

**Figure 1:**
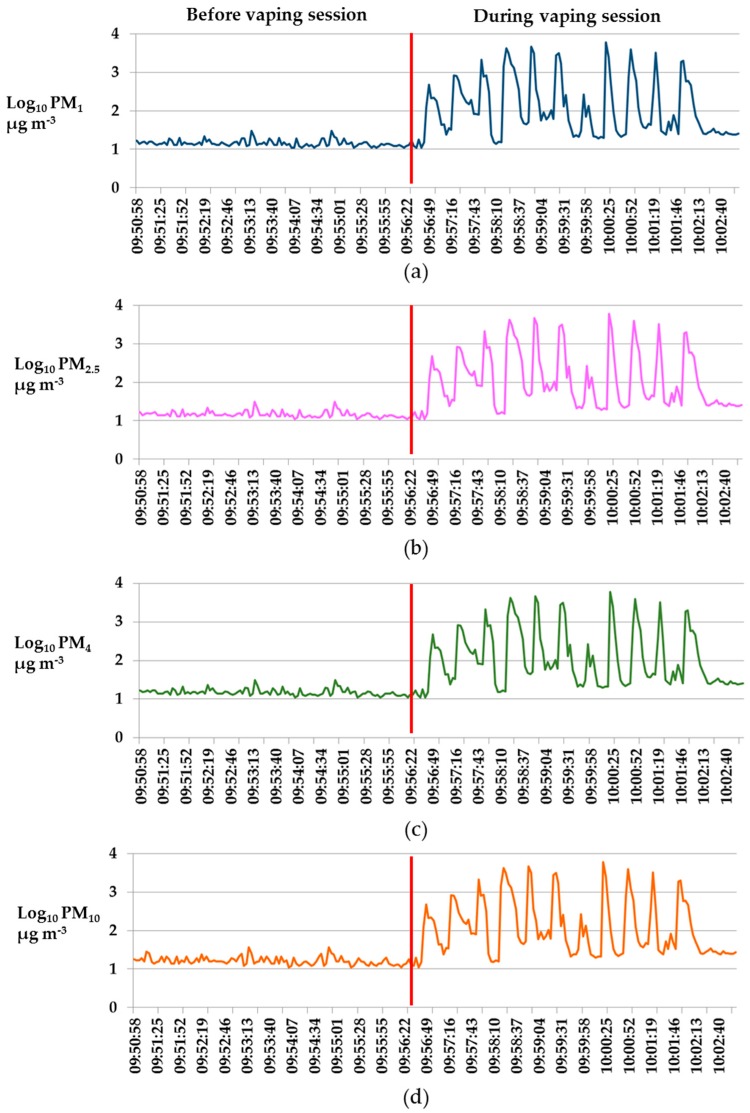
Levels of particulate matter with an aerodynamic diameter smaller than 1 (PM_1_) (**a**), 2.5 (PM_2.5_) (**b**), 4 (PM_4_) (**c**), and 10 µm (PM_10_) (**d**) (values expressed as Log_10_) measured before and during the vaping session of an Iqos1. Red line divided the concentrations measured before and those measured during the vaping session.

In Figure 1 are reported the time series of an experiment.

The aerosol concentrations (μg m^−3^) for PM_10_, PM_4_, PM_2.5_, PM_1_ were measured with 3 s time resolution, using a portable, laser-operated aerosol mass analyzer (Dusttrak ™ II Aerosol Monitor, model 8530, TSI, 0.1–10 µm particle size range). The measurements were carried out in “cumulative” mode, including the mass of all particles smaller than or equal to the defined size. The aerosol was sampled directly through the entry of the instrument without using any tube or collector.

The instrument was placed approximately 1.5 m above the floor level and approximately 1.5 m from the EATC vaper for simulating the breathing zone of a subject exposed to EEV. For each experiment, the measurement was performed from five minutes before until one hour after the end of the vaping session. Twelve puffs were made for each session that lasted about 5.5 min (1 puff each thirty seconds) since the common way of smoking typically consists of 10–12 puffs of a cigarette, for a period of about 5–6 min [20].

The experiments were performed in a dedicated test room of 52.7 m^3^, where temperature and relative humidity during the measurements ranged between 20 and 23 °C and 36% and 40%, respectively. Voluntary smokers (already smokers) were recruited at University of Rome La Sapienza. The study was not sponsored and was approved by the Local Ethics Committee (Policlinico Umberto I/University of Rome La Sapienza, protocol code n. 3520). The air exchange rate (λ) of the dedicated room was calculated using the CO_2_ tracer gas technique, as previously reported [19]. Via linear regression analysis, λ resulted equal to 0.69 h^−1^.

During each experiment, the window and the door were maintained closed. The window and the door were opened after each experiment to reset the room conditions at initial levels of PM.

### 2.2. Statistical Analysis

Statistical elaboration was carried out using IBM SPSS Statistics 25 statistical software (IBM Corp., Armonk, NY, USA). All the statistical analyses were performed on the concentrations of PM_1_ as it resulted the most representative size fraction emitted by the devices tested in the present study (>95% of the total PM emitted in all monitored combinations). Mean and median values were reported to synthetize data distribution for each test combination. In all cases, data distribution was strongly skewed due to the presence of a bottom level and peaks in correspondence of smoke exhalation. Thus, median is more appropriate than arithmetic mean to derive a central tendency since it is much more robust and less sensitive to outliers and peaks. However, we reported also the arithmetic mean and standard deviation for showing the high variability of data. Non-parametric techniques were used to calculate the probability (*p*-value) that the before and after values of each experiment or the levels emitted by each combination EATC/stick or pod were actually the same or not.

## 3. Results

Summary statistic of PM_1_ concentrations recovered, respectively, before and during each experiment (10–12 puff for about 5–6 min) is reported in Table 2.

**Figure 2:**
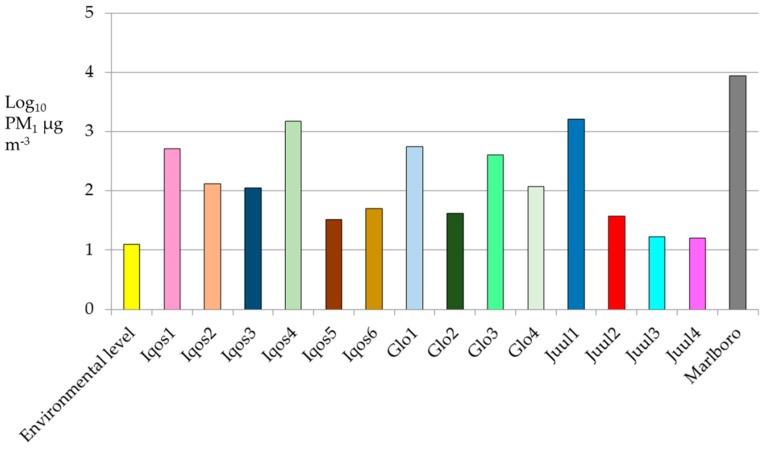
Mean levels of PM with an aerodynamic diameter smaller than 1 µm (PM_1_ expressed as Log_10_PM_1_) measured during each experiment.

Figure 2 shows mean values found for each device and the environmental mean levels of PM_1_ before the experiments.

Data reported in Table 2 and Figure 2 evidence a very high variability of particle loadings both between different devices and with the same device using different sticks/pods. In all the experiments, a statistically significant difference in concentrations of PM_1_ measured, respectively, before and during the vaping/smoking session was found (*p*-value always <0.001).

Moreover, as expected, the highest levels of PM_1_ were measured during the smoking of the traditional cigarette (median value equal to 3430.0 µg m^−3^). With respect to the tested EATCs, PM_1_ concentrations varied widely according to the combination of each different EATC and each different stick or pod, with median values ranging from 11.0 (Iqos3 and Juul2) to 337.5 µg m^−3^ (Iqos4). In general, a relevant worsening of air quality in terms of PM pollution occurred for all the tested combinations. The results of pairwise post hoc tests performed with the Kruskal–Wallis test were reported in Table 3.

Results reported in Table 3 show the high variability in PM_1_ concentrations emitted by different combinations EATC/sticks or pods. In particular, statistically significant differences were found in almost all cases both between different EATCs and between different sticks or pod used with the same EATC.

## 4. Discussion

Over the last few years, the increasing number of EATC users has become a rising concern, so that the use of EATCs is currently considered a global problem for public health. Many threats for human health have been recognized both for active users and subjects exposed to EEV, but further researches are essential to provide more robust evidences on these devices. One of the research agenda is related to the potential pollution determined by the use of these devices in enclosed environments. The present study adds new objective data on indoor air pollution generated by the use of some of the most “famous” and commonly used EATCs.

The first relevant finding of our research is related to the evidence of PM emission during the use of all tested the devices: in all cases, PM concentrations are significantly higher than those measured before the vaping/smoking session. This is partially in line with the results reported previously on some types of e-cigs and IQOS^®^ [2,26,27,28]. Volesky et al. [27], for example, reported a 160- and 103-fold increase of the mean environmental PM_2.5_ concentrations, respectively at 0.5 and 1 m from three different models of e-cig. Ruprecht et al. [28] measured a consistent increase of environmental PM_10_, PM_2.5_ and PM_1_ levels during the smoking session of a conventional cigarette or of an IQOS^®^. In particular, the authors showed that the PM concentration emitted during the use of the IQOS^®^ was substantially lower compared to the level emitted by the conventional cigarette (less than 2% for all the three size ranges). In contrast, the same authors found that the environmental PM concentrations during the use of an e-cig (model “Elips Serie C,” Tank System—Ovale Europe Srl) were not statistically different from the background values; they explained their result assuming that e-cig emissions were mainly partitioned in the vapor phase. Regarding GLO^®^ and JUUL^®^, we did not perform a comparison of our results with other studies because, in our knowledge, this is the first study on environmental levels of PM due to the use of these devices. However, our results confirm that EATCs pollute indoor air, but to a lesser extent than conventional cigarettes, as already demonstrated for other HnBPs by Ruprecht et al. [28]. These findings highlight the need for further studies devoted to evaluating the worsening of indoor air pollution derived from the use of such electronic devices.

The second relevant finding is related to the high variability of particle loadings between tests performed with different devices, but also between the same device used with different sticks/pods. The high variability is likely related both to different sticks/pods used and different way of smoking of each smoker. Indeed, differences in median values found for each device used with different sticks/pods support the consideration on the contribution of different sticks/pods to the different particle loadings, while the high interquartile range resulted for the conventional cigarette should reflect the variability due to the each smoker’s way of smoking.

In addition, we compared our results with the outdoor air PM concentrations recommended by the World Health Organization (WHO) [29]. Indeed, even if no specific values for PM concentrations in private enclosed environments are established, WHO indicates guideline values for the exposure of general population to outdoor air. These values are equal to 50 and 25 μg m^−3^ as average daily environmental levels for PM_10_ and PM_2.5_, respectively. In most cases, we recovered PM values considerably higher than those recommended by WHO, up to 100 time higher for electronic devices and more than 1000 times higher for conventional cigarette. It is worth observing that, during the vaping sessions, the outdoor PM_1_ concentration ranged from 14 to 21 μg m^−3^; therefore, the use of the tested electronic devices with the exception of JUUL^®^, depending on the pod used, determined an indoor air quality markedly worse than the outdoor polluted urban air. This result is of particular concern, also because the size of the aerosol measured was mainly below 1 μm and, therefore, it was capable of penetrating deeply and efficiently into the respiratory system. Moreover, particular harm can be envisaged, since many studies have addressed that airborne particles elicit higher toxicity per unit mass as their sizes decrease [30,31,32].

A particular consideration should be addressed to the great difference observed between aerosol average and median concentrations and to the extremely high values of the arithmetic mean and standard deviations during the smoking/vaping sessions (Table 2). Such occurrence was due to the intense peak aerosol concentration measured upon puff emission: PM_1_ concentrations as high as 22800–46500 μg m^−3^ were measured for very short time intervals (about 12 s). The health effects of short-term exposure to aerosol concentrations have been reported mainly for traffic aerosol and in the time scale of few hours [33,34,35], whereas, as to the intense short term aerosol peak emission from EATC devices, as of now, studies are lacking. This issue is worth of particular attention considering that the aerosol concentrations measured during our experiments address a single smoking/vaping session and a single smoker/vaper, whereas in common indoor environments multiple smoking/vaping sessions and smokers/vapers may be as well encountered. Moreover, it is important to underline that subpopulations such as newborns, children, pregnant women and elderly people may be more vulnerable to such kind of exposure.

The detrimental impact of active—as well as passive—smoking on health is well established, and ongoing efforts at mitigating cardiovascular, pulmonary and cancer risk by enforcing limitations to smoking are providing favorable clinical results [36,37,38]. In keeping with our own findings and other reports linking the use of modified risk products with adverse cardiovascular effects, it is evident that additional efforts are needed to limit the use of JUUL^®^, GLO^®^ and IQOS^®^, as well as other electronic alternatives to smoking, to maximize everyone’s safety [39,40,41].

This study presents some limitations. First of all, we did not perform a systematic assessment of all commercially available EATCs; we evaluated just some of them. However, we selected the most popular devices, both in terms of sales and international diffusion; consequently, our findings add relevant information on indoor air pollution determined by these devices. Moreover, other e-cigs and HnBPs were already tested previously, while, in our knowledge, GLO^®^ and JUUL^®^ have never been studied in terms of environmental PM air pollution generated by their use. Second, we resorted to volunteers and, thus, our results were probably influenced in part by the individual way of smoking/vaping. On the other hand, the smoking session performed by a real smoker allows to measure in the ambient air the aliquot of PM really emitted by a user (thus not including the amount retained in her/his respiratory tract), and not the total PM generated by the device when smoked by a smoking machine. However, for a complete evaluation of the environmental risk related to the EATC emissions, the chemical and size distribution aerosol characterization, together with the relevant aerosol respiratory dosimetry of the passively exposed subjects will have to be carried out.

## 5. Conclusions

The present study provides data on indoor air PM pollution derived from the use of some of the most commonly used EATCs, among those JUUL^®^ and GLO^®^ were evaluated for the first time. The results demonstrated that all tested EATCs worsen indoor air quality during their use. Thus, specific legislative measures are needed in order to regulate the use of these devices in public indoor environments, while educational interventions are necessary for increasing the perception of the vapers/smokers about the risks linked to the indoor use of these devices for both active and passive smokers.

## Figures and Tables

**Table 1:** Devices, flavors and relative codes of the experiments.

EATC Types	Stick/Pod	Code
IQOS^®^	Heets Amber label	Iqos1
	Heets Blue label	Iqos2
	Heets Bronze label	Iqos3
	Heets Sienna label	Iqos4
	Heets Turquoise label	Iqos5
	Heets Yellow label	Iqos6
GLO^®^	Neo Aegean stick	Glo1
	Neo Beryl stick	Glo2
	Neo Ultramarine stick	Glo3
	Neo Yellow stick	Glo4
JUUL^®^	Golden Tobacco	Juul1
	Mango	Juul2
	Mint	Juul3
	Royal Creme	Juul4
Marlboro^®^ gold	–	Marlboro

**Table 2:** Air levels of PM with an aerodynamic diameter smaller than 1 µm (PM_1_; μg m^−3^) before and during each experiment.

Experiment Code	Before Experiment		During Experiment (10–12 Puff for about 5–6 min)		*p*-Value (Calculated Probability)
	Arithmetic Mean [Standard Deviation]	Median [Interquartile Range]	Arithmetic Mean [Standard Deviation]	Median [Interquartile Range]	
Iqos1	14.2 [3.7]	13.0 [3.0]	517.7 [1048.9]	70.0 [383.0]	<0.001
Iqos2	14.6 [6.5]	13.0 [2.0]	132.0 [619.3]	21.0 [12.0]	<0.001
Iqos3	9.2 [1.5]	9.0 [2.0]	112.3 [469.4]	11.0 [5.0]	<0.001
Iqos4	9.1 [5.8]	7.0 [1.0]	1511.3 [2707.2]	337.5 [1568.0]	<0.001
Iqos5	23.7 [4.6]	22.0 [6.0]	32.4 [8.4]	32.0 [16.0]	<0.001
Iqos6	10.7 [2.1]	10.0 [2.0]	49.8 [128.8]	21.0 [19.0]	<0.001
Glo1	8.8 [2.4]	8.0 [2.0]	552.6 [1477.5]	66.0 [205.5]	<0.001
Glo2	8.7 [0.9]	8.0 [1.0]	42.0 [115.1]	13.0 [9.0]	<0.001
Glo3	26.9 [1.7]	27.0 [2.0]	406.5 [1335.6]	31.0 [101.0]	<0.001
Glo4	12.1 [1.9]	12.0 [2.0]	117.7 [336.8]	23.0 [38.0]	<0.001
Juul1	8.3 [2.3]	7.5 [1.0]	1637.9 [6387.6]	110.0 [289.0]	<0.001
Juul2	10.9 [1.5]	10.0 [1.0]	37.7 [208.3]	11.0 [3.0]	<0.001
Juul3	13.8 [1.9]	13.0 [4.0]	16.7 [5.4]	15.0 [4.0]	<0.001
Juul4	13.3 [1.5]	13.0 [2.0]	16.0 [5.0]	15.0 [4.0]	<0.001
Marlboro	3.9 [1.2]	4.0 [1.0]	8638.5 [1,2215.9]	3430.0 [8620.0]	<0.001

**Table 3:** Pairwise post hoc tests between the levels of PM with an aerodynamic diameter smaller than 1 µm (PM_1_; μg m^−3^) measured in each experiment.

	Iqos2	Iqos3	Iqos4	Iqos5	Iqos6	Glo1	Glo2	Glo3	Glo4	Juul1	Juul2	Juul3	Juul4	Marlboro Gold
**Iqos1**	*	*	*	*	*	*	*	*	*	*	*	*	*	*
**Iqos2**		*	*	*	*	*	*	*	*	*	*	*	*	*
**Iqos3**			*	*	*	*	ns	*	*	*	ns	ns	ns	*
**Iqos4**				*	*	*	*	*	*	*	*	*	*	*
**Iqos5**					*	*	*	*	*	*	*	*	*	*
**Iqos6**						*	*	*	*	*	*	*	*	*
**Glo1**							*	*	*	*	*	*	*	*
**Glo2**								*	*	*	ns	ns	ns	*
**Glo3**									*	*	*	*	*	*
**Glo4**										*	*	*	*	*
**Juul1**											*	*	*	*
**Juul2**											*	*	*	*
**Juul3**												*	*	*
**Juul4**													*	*

* = *p*-Value (calculated probability) <0.05; ns = not significant: *p*-Value (calculated probability) >0.05.
